# Nonsexual Transmission of Anogenital Warts in Children: A Retrospective Analysis

**DOI:** 10.1100/tsw.2007.276

**Published:** 2007-11-26

**Authors:** Valerie Jones, Shawn J. Smith, Hatim A. Omar

**Affiliations:** Division of Adolescent Medicine, Department of Pediatrics, University of Kentucky, Lexington, KY 40536, USA

**Keywords:** human papilloma virus, anogenital warts, sexual abuse, prenatal transmission, abnormal pap smears

## Abstract

The purpose was to evaluate the prevalence of sexual abuse in patients who were referred to a pediatric gynecologist for evaluation based on the clinical findings of anogenital warts. A retrospective analysis was performed on 131 patients between the ages 6 month and 9 years referred to a pediatric gynecologist after the finding of anogenital warts by a clinical provider, parent or caregiver. A complete physical examination under colposcopy by a the same, trained pediatric gynecologist was completed, and a complete medical and family history including maternal and sibling history for evidence of Human Papillomavirus (HPV) and anogenital warts. The legal system completed a full investigation to examine the sexual abuse allegations. In 131 patients with anogenital warts, a maternal history of warts, cervical dysplasia or both was present in 66 (50%). The remaining patients had either a negative maternal history for HPV clinical findings (54 patients or 41.2%), or maternal history was unknown (11 patients, or 8.3%). Of 131 patients, 81 (61%) patients had a sibling. Of those with siblings 40 (49.4%) had warts and 41 (50.6%) did not. Forty-five (34%) of the cases had a positive maternal history for warts, dysplasia or both but also had a sibling. In that cohort, 32 (71%) of the siblings also had anogenital warts. Three of 131 patients were ruled suspicious for sexual abuse by the legal authorities but not confirmed. Of those three patients two were female and one was male. Two had no maternal history for HPV and both of these patients had a sibling without anogenital warts. Most cases of anogenital warts in children are likely to be the result of non-sexual transmission, namely prenatal mode. Thus, these patients should be handled differently by the legal system unless other reasons for suspicion exist. This study also showed the importance of maternal gynecologic history.

## INTRODUCTION

Human papillomavirus (HPV) is extremely prevalent and induces a wide spectrum of clinical disease. It is estimated that 20 million Americans are currently infected with HPV and more than 5.5 million new cases are diagnosed annually[1]. HPV is a DNA virus that can manifest as an asymptomatic, self-limiting infection but also as a malignancy. It infects the genital skin and mucosa including the vaginal tract, cervix and anal canal. HPV anogenital infection is the most common sexually transmitted infection in the United States. It occurs in more than 40% of sexually active adolescent females[2].

Cancers attributed to HPV include the cervix, anus, vulva/vagina, penis, mouth and oropharynx. Specifically cervical cancer is the second most common malignancy among females worldwide. There are approximately 500,000 newly diagnosed cases each year and about 275,000 deaths annually[3,4]. There are over 100 identified types of HPV, but approximately 40 types can infect the genital tract. High Risk types are those HPV types found more frequently in cervical cancer. These include HPV-16, HPV-18, HPV-31, and HPV-45[5].

Recognizing the variability of modes of transmission, especially the nonsexual mode of HPV infection is especially important in the pediatric population. Pediatric gynecologists receive many referrals for sexual abuse evaluation based on clinical findings of anogenital warts. We present our data to further emphasize that the presence of anogenital warts in the pediatric population can be obtained from various modes of transmission. Before assuming sexual contact as the means of acquisition, it is essential to place physical findings in the context with suspicion, history and interview. This will prevent families from undergoing an unnecessary, and psychological damaging investigation that may be based on assumptions.

## METHODS

This study is a retrospective chart review of medical records of prepubertal patients, ages 6 months to 9 years of age referred to a Pediatric Gynecology clinic for evaluation of possible sexual abuse due to the findings of anogenital warts. A parent, caregiver, or practitioner noted the physical finding. The patients were evaluated between January 1, 2005 and December 12, 2006.

All patients were included and there were no exemptions. A careful physical examination under colposcopy by the same trained pediatric gynecologist was completed. Following this, information was obtained including maternal and sibling history. The legal system completed a full investigation to examine the sexual abuse allegations.

## RESULTS

Our study consisted of 131 patients (see Table 1). Patients were referred from all regions in the state of Kentucky to a pediatric gynecologist in an academic center. Of 131 patients, 23 (17.5%) were male and 108 (82.5%) were female. 103 (78.6%) were white, 27 (20.6%) were black and 1 (0.76%) was Hispanic.

In 131 patients with anogenital warts, a maternal history of warts, cervical dysplasia or both was present in 66 (50%). The remaining patients had either a negative maternal history for HPV clinical findings, which included 54 patients (41.2%), or maternal history was unknown (11 patients, or 8.3%).

This review also evaluated siblings of the patients. In this population 81 (61%) patients had a sibling. Of those with siblings 40 (49.4%) had warts and 41 (50.6%) did not.

Forty-five (34%) of the cases had a positive maternal history for warts, dysplasia or both but also had a sibling. In that cohort, 32 (71%) of the siblings also had anogenital warts.

Finally after careful physical exam, review of the patients' history, and a detailed interview via the legal system, three of 131 patients were ruled suspicious for abuse. Of those three patients two were female (30 months of age and 7 years of age) and one was male (34 months of age). Two had no maternal history for HPV and both of these patients had a sibling without anogenital warts. None of the cases was judged definite for abuse.

**Table 1. Characteristics of the Patient Population.**

| | | | |
|---|---|---|---|
| **Gender** | Male 23 (17.5%) | Female 108 (82.5%) | |
| **Ethnicity** | White 103 (78.6%) | Black 27 (20.6%) | Hispanic 1 (0.76%) |
| **Siblings** | Yes 81 (61%) | No 50 (39%) | |
| **Sibling History** | Positive for Anogenital Warts 40 (49.4%) | | Negative for Anogenital Warts (50.6%) |
| **Maternal History** | Anogenital Warts 15 (11.4%) | | Abnormal Pap Smear 43 (32.8%) |
| | Unknown History 11 (8.4%) | | Warts& Abnormal Pap Smear |
| | Negative History 54 (41.2%) | | |

## DISCUSSION

Human papillomavirus is extremely prevalent. HPV infection is one of the most common sexually transmitted infections (STIs) of the anogenital tract. Molecular diagnostic techniques have demonstrated the presence of HPV in 11% to 80 of asymptomatic sexually active young women[6,7]. While the transmission of HPV in the adult population is well described, the transmission and prevalence of HPV in the pediatric population remains poorly described. Before 1990 only 74 cases of condyloma acuminata were reported[8]. The increasing incidence of anogenital warts in children is most likely due to increasing infection of anogenital infection in adults[9]. In the past, the finding of HPV anogenital infection caused the practitioner to pursue an investigation for sexual abuse. However, in the last decade there has been increasing evidence of nonsexual transmission of HPV in this population. The acquisition of HPV can occur in a variety of ways. Modes of transmission include directly from personal contact or indirectly through contaminated objects or surfaces[10]. Maternal transmission has also been a well-described mode of viral spread. Specifically, laryngeal papillomas occurring in children and adolescents have been proposed to be linked to maternal genital condylomata[11]. Also, HPV DNA has been isolated in nasopharyngeal aspirates of infants born by vaginal delivery to mothers with genital HPV infection[12]. HPV DNA has also been identified in amniotic fluid and within the placenta suggesting hematogenous spread[13,14,15]. More recently familial transmission has been described suggesting the father as a possible reservoir for transmitting the virus[16].

A review of the various modes of acquisition of HPV and anogenital warts in children between newborn and 12 years by Syrjanen and Puranen showed a high prevalence of clinical disease obtained by other means of transmission than sexual contact. In a total of 185 patients with anogenital warts and a likely known source of acquisition, 67 (36%) infections were obtained through autoinoculation, non-sexual transmission from other family members and possible vertical transmission from the mother[10]. Furthermore, a study published in 2006 by Beznos et. al. illustrated the prevalence of vulvar papillomatosis in female adolescents without previous sexual history. In a cohort of 44 females between the ages 12-18 years, 20 (45%) tested positive for HPV, and denied any intimate sexual contact, prior history of genital tract condylomata or sexual abuse[17].

The awareness and perception of obtaining HPV through nonsexual contact is especially important when we consider the implications of a sexual abuse investigation. From a historical standpoint, the American Academy of Pediatrics Committee on Child Abuse and Neglect, “Guidelines for the evaluation of sexual abuse of children,” stated that sexual abuse is “probable” in a child with postnatal condylomata acuminata. In a review article by Gutman et al (1992), the authors illustrated the interesting historical perspective that in the 1990’s gonorrhea was thought to be transmitted via nonsexual contact and it was not until much later that pediatric gonorrhea became clinical evidence of sexual abuse[18].

Furthermore, in a study by Cohen et al. (1991) 73 children under the age of three years with anogenital warts were examined for sexual abuse during a 2-year period. No evidence of sexual abuse was detected in 66 of the 73 children[19].

In our study, the largest published number of patients referred for evaluation of sexual abuse due the single finding of anogenital warts, suspicion for sexual abuse was reported in only 3 of 131 patients or 2%. This evidence illustrates that clinical evidence of HPV does not usually correlate with sexual abuse. We recognize that this is a retrospective study and including the types of HPV may provide further insight into characterizing the types of HPV specific modes of transmission. This study demonstrates that anogenital warts alone are not an indication for sexual abuse investigation and all aspects of the history, physical exam and interview are necessary. In doing so, we can avoid the unnecessary psychological impact these investigations have on patients and families. It also shows that possible prenatal transmission is not unusual and that other studies are needed to determine the prevalence of prenatal transmission of other HPV type that may not be clinically evident, but may cause more severe problems such as cervical cancer later in life.
